# Looking to Deceive: Voluntary Gaze Control Reduces the Discriminative Power of Eye-Movement-Based Concealed Information Testing

**DOI:** 10.3390/jemr19050096

**Published:** 2026-09-02

**Authors:** Nikola Milosavljević, Miloš Panjevac, Milica M. Janković, Vanja Ković

**Affiliations:** 1 Faculty of Special Education and Rehabilitation, University of Belgrade, Visokog Stevana 2, 11000 Belgrade, Serbia; 2 School of Electrical Engineering, University of Belgrade, Bulevar Kralja Aleksandra 73, 11120 Belgrade, Serbia; milospanjevac@yahoo.com (M.P.); piperski@etf.bg.ac.rs (M.M.J.); 3 Faculty of Philosophy, University of Belgrade, Čika-Ljubina 18-20, 11000 Belgrade, Serbia; vanja.kovic@f.bg.ac.rs

**Keywords:** eye-tracking, deception detection, concealed information test, recognition memory, voluntary gaze control, oculomotor control, mock crime, machine learning

## Abstract

The Concealed Information Test (CIT) detects whether an individual recognizes crime-relevant information, and eye tracking has been proposed as a non-invasive method for detecting this recognition. It remains unclear, however, whether individuals who are motivated to conceal their knowledge can voluntarily control their gaze and thereby undermine the test’s validity. We developed a novel free-viewing, parallel-presentation eye-movement-based CIT and administered it to 75 participants randomly assigned to three groups: Innocent, Informed Innocent, and Guilty. For each participant, twelve eye-tracking measures were calculated as differential (crime-relevant minus control) scores and analyzed using four supervised classifiers under a participant-wise, leave-two-out cross-validation, with accuracy reported at both the trial and participant levels. Cumulative dwell- and duration-based measures, together with glance and fixation counts, carried most of the discriminative signal. The classifiers demonstrated high accuracy when distinguishing between the naive and knowledgeable but non-concealing individuals, as well as between knowledgeable non-concealing individuals and culpable individuals. However, classification performance fell to chance-level probability when distinguishing naive and culpable individuals. Thus, participants who were motivated to avoid detection markedly reduced their distinctive eye-movement signature with voluntary gaze control, becoming indistinguishable from truly naive individuals. We discuss methodological implications for designing eye-movement-based CITs that exhibit greater resilience against voluntary gaze modulation.

## 1. Introduction

Establishing whether an individual holds knowledge that they are attempting to hide is a central problem in forensic practice. The Concealed Information Test (CIT), originally introduced as the Guilty Knowledge Test [1,2], was developed to address this problem. Instead of evaluating whether an individual is lying, the CIT presents multiple-choice questions where one critical item (the real detail from the crime; i.e., the probe) is hidden among several plausible but crime-irrelevant alternatives chosen such that an innocent individual cannot distinguish them from the critical item. Consistently enhanced responses to the probe indicate that the individual recognizes it and therefore possesses information that only a knowledgeable individual would have.

Compared to other detection paradigms, the CIT possesses a well-established theoretical foundation, substantial empirical validity evidence, and minimizes the risk of falsely classifying innocent suspects [3,4]. Meta-analyses of autonomic measures (most notably the skin conductance response, together with respiration and heart rate) and of the P300 event-related potential report large effect sizes and high discrimination between knowledgeable and naive examinees [4,5,6]. Theoretically, responses to crime-relevant items are driven by two distinct mechanisms: an orienting response triggered by personal significance of the item and arousal- and inhibition-related processes engaged when an individual attempts to withhold critical knowledge and suppresses recognition. These mechanisms are partially dissociable and appear to drive different physiological measures [7,8]. The CIT is currently established as the most scientifically validated paradigm for memory-based deception detection.

A persistent threat to the CIT, however, is the use of deliberate strategies by examinees attempting to evade detection. Autonomic CITs can be substantially weakened when knowledgeable examinees perform covert physical or mental manipulations during neutral items [9], and early P300 protocols proved similarly vulnerable [10]. These findings motivated the development of more manipulation-resistant procedures, such as the P300-based Complex Trial Protocol (CTP), which was explicitly designed to detect or withstand such strategies [11]. The general lesson is that any measure under partial voluntary control can, in principle, be modulated by a motivated examinee and that the robustness of a detection method cannot be assumed but must be tested directly.

Against this background, eye tracking has emerged as a promising channel for memory detection. In addition to being less invasive and allowing the simultaneous presentation of multiple stimuli, eye movements provide a more direct index of cognitive processing, exhibit shorter response latencies, and are less susceptible to individual variability than traditional autonomic measures [12]. Accumulating evidence indicates that eye movement patterns can reveal prior exposure and memory. For example, familiar faces and scenes are scanned with fewer and more focused fixations than novel ones, and these eye-movement memory effects can arise even in the absence of explicit recognition [13]. In the context of deception detection, various oculomotor metrics are utilized to reveal concealed, crime-related memories in mock-crime paradigms. Specifically, fixation-related (e.g., number and duration of fixations) [14], saccade-related (e.g., oculomotor inhibition) [15], blink-related (e.g., decreased blink rate) [16], pupil-related (e.g., increased pupil dilation) [17], and scanpath measures (e.g., altered gaze transitions and exploration patterns) [18] have all demonstrated the capacity to successfully differentiate recognized from unrecognized items.

Researchers are increasingly working to map out exactly when and why eye-movement-based CITs work and under what specific conditions they reliably detect concealed recognition, focusing on factors such as task instructions (free viewing vs. memorization vs. search), stimulus layout (sequential vs. simultaneous presentation), type of stimuli (faces, objects, words), and presence or absence of countermeasures. Gaze behavior toward familiar faces distinguishes knowledgeable from naive viewers [19], and the magnitude of these effects depends strongly on the task that participants perform while viewing, with short-term memory tasks yielding more diagnostic gaze behavior than simple detection tasks [20]. Empirical evidence indicates that distinct deceptive intentions produce distinguishable oculomotor behavior, whereby unique fixation patterns distinguish acts of concealment from those of response fabrication [21]. Alongside this, a recent investigation distinguished two sources of deception-related changes in gaze (an involuntary component, reflecting deception-induced cognitive load, and a deliberate component, reflecting voluntary gaze modulation) and demonstrated that their balance and even the direction of fixation effects shifts depending on how stimuli are displayed (sequential versus simultaneous display) [22]. Reviews of the current literature highlight the substantial potential of eye-movement-based CIT for memory detection applications, while cautioning that its resistance to deliberate gaze control is not yet well established, and further research is needed [23].

The extent to which eye movements can be voluntarily modulated within eye-movement-based CIT frameworks remains a critical, yet largely unresolved question. Oculomotor behavior is not merely reflexive; rather, the visual system supports robust top-down control. This capacity is clearly demonstrated by an individual’s ability to suppress reflexive saccades and voluntarily direct or maintain fixation, as evidenced in paradigms such as the antisaccade task [24]. If an individual who recognizes the probe can voluntarily suppress or alter the oculomotor response typically elicited by such recognition, the discriminative power of an eye-movement-based CIT could be significantly reduced. While existing demonstrations of oculomotor control have predominantly relied on facial stimuli or externally imposed, rule-based scanning instructions, empirical understanding remains limited regarding spontaneous gaze regulation. Specifically, far less is known about how motivated concealment influences free-viewing behavior toward object stimuli within realistic mock-crime scenarios.

The present study addresses this question by implementing a newly developed eye-movement-based CIT, wherein crime-relevant, control, and neutral object images are presented simultaneously and viewed freely. To separate the contribution of crime-relevant knowledge from the motivation to avoid detection, we compared three distinct experimental groups: an Innocent group, with no knowledge of the crime-relevant details, only aware that a crime had been committed; an Informed Innocent group, who memorized the crime-related knowledge but did not execute the mock crime and thus viewed the eye-movement-based CIT stimuli under explicit instructions to respond naturally without concealment; and a Guilty group, who committed the mock crime and were explicitly instructed to conceal recognition of critical items to avoid detection during the eye-movement-based CIT. Including an Informed Innocent group is also important from an ecological perspective, as crime-relevant details can inadvertently leak to genuinely innocent individuals who may then exhibit recognition-like gaze patterns despite their lack of involvement in the crime [25]. This design allows the effect of crime-relevant knowledge alone (Innocent versus Informed Innocent) to be distinguished from the effect of motivated concealment (Informed Innocent versus Guilty). The objectives of the present study were exploratory in nature: while the initial goal was to identify which eye-tracking measures discriminate among the experimental groups, the primary objective was to assess whether participants motivated to avoid detection can systematically reduce their detectability relative to equally knowledgeable, but non-concealing participants. Rather than prioritizing classification accuracy, we investigated whether motivated guilty participants can voluntarily suppress recognition-related gaze behavior patterns during free-viewing to such an extent as to render themselves statistically indistinguishable from innocent participants. Therefore, this study shifts the empirical focus from whether eye-tracking paradigms can detect concealed knowledge to whether their diagnostic validity persists under deliberate, voluntary gaze suppression. The resulting findings are interpreted in terms of their methodological implications for designing eye-movement-based CITs that exhibit greater resilience against voluntary gaze modulation.

## 2. Materials and Methods

### 2.1. Participants

Seventy-five second-year undergraduate psychology students (58 female, 17 male; mean age = 20.21 years, SD = 0.47, range 20–22) with normal or corrected-to-normal vision from the University of Belgrade participated in the study in exchange for course credits. They were unfamiliar with eye-tracking interfaces, as this experiment represented their first exposure to an oculomotor recording setup. Participants were randomly assigned to one of three groups: *Innocent*, *Informed Innocent*, and *Guilty*. Each group consisted of 25 participants.

### 2.2. Mock Crime Scenario

The mock crime scenario was designed to resemble the real-life crime situation as much as possible. Participants assigned to the *Guilty group* were given a background story with step-by-step instructions on how to commit theft. The scenario included an explicit description of the motivation for committing the crime, and a request for the participants to enter a professor’s office and steal the envelope with checks. For relevant items, we chose objects that were directly related to the crime and had been in physical contact with the perpetrator, since the central details of the crime are more likely to be remembered than the peripheral ones, according to previous studies [26,27,28]. Items in eye-movement-based CIT were chosen to be pictorial because images are better remembered than words [29]. In order to be as realistic as possible, images were created by taking photos of objects from the mock crime scene and of real objects in the faculty building. We carefully selected control and neutral items to ensure that crime-relevant items could be easily distinguished from them. While the individual visual stimuli within each crime-relevant category were matched for visual and cognitive complexity, these parameters varied across different categories of crime-relevant items.

A pilot study was conducted to make sure that our mock crime procedure worked properly and to check whether the instructions were sufficiently clear and whether the participants could successfully carry out the theft based on them, as well as to check the appropriateness of chosen control and neutral items. The sample consisted of 16 psychology students (12 females). Participants were presented with a list of items and asked to guess which of them were the critical details from the theft. Then they performed the mock crime and answered pre-determined open-ended questions. Twelve participants successfully committed the mock crime, which suggested that it was necessary to replace one crime-relevant item and to make certain adjustments to the experimental setting (e.g., hiding objects in the purse made it impossible for some participants to locate them). We set a time limit of 6 min, based on the median of time that was required to commit the theft successfully. The selected control and irrelevant items proved to be appropriate—the participants could not guess which item was related to the theft, with choice being at the level of the guessing probability. The mock crime scenario was corrected and simplified in accordance with the feedback we received from the participants.

### 2.3. Eye-Movement-Based CIT Design

**Figure 1:**
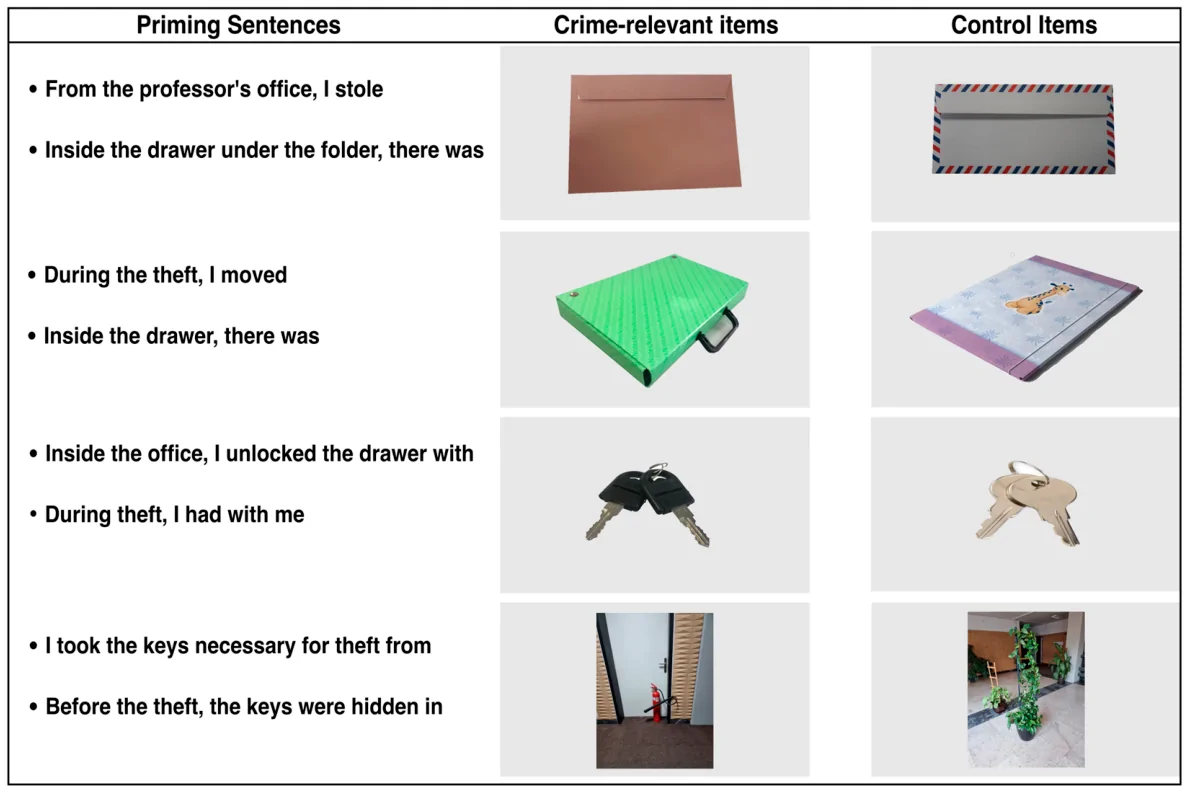
Priming sentences with corresponding crime-relevant and control items.

We aimed to develop a new eye-movement–based paradigm for detecting concealed knowledge. This novel experimental test examines whether the individual recognizes crime-relevant items. It was hypothesized that knowledgeable participants (both the *Informed Innocent* and the *Guilty*) would demonstrate recognition of specific crime details, manifested as distinct response patterns to critical versus control stimuli, whereas innocent participants were expected to exhibit uniform responding across stimulus conditions due to a lack of crime-specific knowledge. For each of four crime-relevant items (envelope, folder, keys, location), two priming sentences in the first-person singular were created: one referring to the perpetrator’s action during the crime (e.g., “From the professor’s office, I stole”) and the other pertaining to the item’s location during the crime (e.g., “Before the theft, the keys were hidden in”). As illustrated in Figure 1, one corresponding control item was developed for each of the four crime-relevant items (totaling four control images). In addition, two sets of three neutral images were created per crime-relevant item, resulting in 24 neutral images. Therefore, eye-movement-based CIT consisted of eight priming sentences and 32 images of items (four crime-relevant items × eight images: 4 crime-relevant, 4 control and 4 × 6 neutral images). For the complete list of images used in our eye-movement-based CIT, see Supplementary Images S1.

Control and neutral items shared the same object categories as the crime-relevant items but were entirely unrelated to the crime. Control items were presented in the same manner as crime-relevant items: each one was displayed on the screen simultaneously with one set of three neutral items. Consequently, in half of the trials, participants viewed a parallel display of four images consisting of one crime-relevant item and three neutral items and in the other half containing one control item and three neutral items. The presentation of both crime-relevant and control items was fully counterbalanced. These stimuli appeared with equal frequency across the four screen quadrants, thereby eliminating any potential confounding effects related to image positioning. For instance, prior research [30] has demonstrated that item positioning significantly influences eye-tracking metrics; specifically, adults exhibit an upper-left bias during initial saccades, which directly impacts both Entry Time and Time to First Saccade.

There were a total of 32 trials—four crime-relevant item categories × two priming sentences × two crime-relevant/control conditions x two sets of neutral items. The trial order was quasi-randomized and remained identical across all participants, due to the absence of a built-in trial randomization feature within the SMI Experiment Center 3.7 software (SensoMotoric Instruments, Teltow, Germany). Participants were given these instructions:

*“This test is being conducted to determine whether you were involved in the theft that occurred in the professor’s office.*

*Your every eye movement will be continuously tracked and recorded throughout the test.*

*Just as in the practice session, a sentence will first be presented on the screen. Your task is to press the “SPACE” bar as soon as you have silently read the sentence.*

*Following this, four images will be displayed on the screen. Only one of these images correctly completes the previously shown sentence. Your task is to observe what is presented on the screen. After a while, the images will automatically disappear from the screen.”*

Each trial began with (1) a blank screen on which the central fixation point was presented for a randomly selected duration of time that varied between 500 ms and 1000 ms, after which (2) the priming sentence appeared at the center of the screen. Participants read the priming sentence silently, and pressed the SPACE key as soon as they had read it. Again, (3) the blank screen with a central fixation point was presented for a random duration of time, between 500 ms and 1000 ms. This variable blank-screen presentation interval was used to render the onset of the four-image display temporally unpredictable, thereby reducing stimulus anticipation and discouraging anticipatory or preparatory eye movements. This was intended to ensure that gaze recorded at image onset more closely reflected stimulus-driven processing rather than entrained timing. A central fixation point served to facilitate central refixation before each display, ensuring that all images were presented from a common initial gaze position. (4) Four images then appeared simultaneously on the screen for 5000 ms, and participants were instructed simply to look at the images while they were on the screen. Afterwards, a (5) blank screen was presented for 1000 ms.

### 2.4. Procedure

Each session began with a condition-specific phase, after which all participants immediately completed the same eye-movement-based CIT developed specifically for this study. Participants assigned to the *Guilty* condition first read the background story and retained step-by-step mock crime instructions, then committed a mock crime which involved stealing an envelope with checks from a professor’s office (therefore, they held first-hand, episodic knowledge of all the crime-relevant items). After committing the theft, they handed the stolen items to a research assistant and then were instructed to conceal their involvement in the crime and to avoid detection, without receiving any explicit guidance on how to do so or which concealment strategies to use. Participants from the *Informed Innocent* group read a newspaper article containing images of all the crime-relevant items alongside a detailed description of the theft (i.e., they acquired the same crime-relevant knowledge as the *Guilty* group); their recall of these details was verified prior to testing. Finally, *Innocent* participants read a newspaper article describing only the publicly known, general details of the theft that occurred at the faculty, and they were naive to any crime-relevant information.

Participants were tested individually in the Laboratory for Neurocognition and Applied Cognition at the University of Belgrade. The eye movements were recorded using an SMI RED-m portable remote eye-tracker (SensoMotoric Instruments, Teltow, Germany, sampling rate: 60 Hz) mounted below a 24-inch monitor (resolution: 1920 × 1080) on which the stimuli were presented. The viewing distance was held constant across all participants at approximately 60 cm. The stimuli were presented using SMI Experiment Center 3.7 software; raw data were collected with SMI iView RED-m software 3.7 (SensoMotoric Instruments, Teltow, Germany). A five-point calibration was performed for each participant before the task, followed by a validation step, and the experiment was initiated only after the calibration met the predefined acceptance criterion. Calibration was accepted only if the average spatial accuracy error was below 0.5° of visual angle across all calibration points; if this criterion was not met, calibration was repeated. Data quality was further assessed by visual inspection of each recording in the SMI BeGaze software 3.7 (SensoMotoric Instruments, Teltow, Germany) immediately after acquisition and by the per-participant tracking ratio. To minimize systematic gaze drift due to head movement, head position was stabilized with a chin rest, and central fixation markers presented between blocks allowed gaze drift to be monitored across the session. Participants with pervasive tracking loss or a recording that failed visual inspection would have been excluded; in practice, all participants had a mean tracking ratio above 80% and passed inspection, so no participant was excluded from the analyses. Each participant completed a set of practice trials to familiarize themselves with the experimental protocol. The practice trials utilized a distinct set of priming sentences and images pertaining to the academic faculty the participants attended. During the testing phase, each participant viewed 32 four-image displays. In half of these trials, one image correctly completed the preceding sentence (relevant stimulus condition); in the remaining half, none of the images completed the sentence correctly (control stimulus condition). The experimental session lasted for approximately 30 min in total.

### 2.5. Eye-Tracking Measures and Data Analysis

Twelve eye-tracking metrics were extracted for each four-image screen per participant using BeGaze software. These metrics included: Entry Time, Net Dwell Time [%], Dwell Time [%], Glance Duration, Diversion Duration, First Fixation Duration, Glances Count, Revisits, Fixation Count, Fixation Time [%], Average Fixation Duration, and Time to First Saccade. These twelve metrics represent the standard area-of-interest (AOI) outputs of the BeGaze software and were deliberately retained in full. This allowed the classifiers, rather than our a priori assumptions, to determine which measures carried the recognition signal for each group contrast. Crucially, redundancy among correlated features was not ignored; it was handled empirically within the modeling pipeline rather than through preliminary feature selection. Pupillometry metrics were excluded from analysis due to an inability to control and keep constant ambient lighting conditions, monitor illumination and contrast, and maintain stimulus brightness. Table 1 provides, for each of the twelve metrics, an operational definition and the cognitive process it is most commonly considered to index. In general, recognition and heightened stimulus salience are expected to elicit earlier initial orienting, longer sustained gaze, and more frequent re-inspection of the recognized item. Accordingly, participants with crime knowledge should produce larger positive differential (crime-relevant minus control) scores than naive innocents across latency-, duration-, and count-based measures, thereby providing a basis for distinguishing the groups. These metric–process correspondences should be read as interpretive heuristics rather than one-to-one mappings: a given oculomotor measure can reflect more than one process depending on task demands, and the present paradigm was not designed to isolate these processes.

Each participant viewed 32 trials (16 crime-relevant and 16 control stimulustrials). There were a total of 2400 four-image screens viewed by participants (75 participants × 32 trials). For each participant, the 32 trials were paired based on the spatial position of the relevant and control items within the four-image display. Differential measures for each eye-tracking metric were subsequently calculated by subtracting values of the four-image screen with a control item from those of the four-image screens with relevant items. The subtraction process yielded a final dataset of 1200 differential trials. In total, 800 differential trial data points were available as the input to the three binary classification tasks (16 differential trials × 50 participants), and each task was addressed with four supervised algorithms: k-Nearest Neighbors (KNN), Random Forest (RF), Logistic Regression (LR), and Support Vector Machine (SVM). Prior to model training, any missing feature values in cases where a metric could not be computed for a trial were handled within each cross-validation fold using mean imputation, and the z-score normalization was applied. Across the three classification datasets, missing values represented a small proportion of the overall feature matrix: 1.31% of cells for *Innocent* vs. *Informed Innocent* (220 of 16,800), 1.59% for *Innocent* vs. *Guilty* (267 of 16,800), and 1.24% for *Informed Innocent* vs. *Guilty* (209 of 16,800). Missing values were concentrated in Time to First Saccade, which was unavailable for 14.9–18% of trials most likely because no qualifying saccade was detected. Entry Time and Revisits were each affected in 3.6–5.1% of trials, whereas the remaining metrics were essentially complete. Given that Time to First Saccade demonstrated one of the smallest univariate group effects and imputation was strictly restricted to the training folds, it is unlikely that missing data substantially affected the classification results. Three separate classification tasks were performed: *Innocent* vs. *Guilty*, *Innocent* vs. *Informed Innocent*, and *Informed Innocent* vs. *Guilty*. Four supervised classification algorithms, KNN, RF, LR, and SVM, were trained on the 12 differential (crime-relevant minus control) eye-tracking features. Hyperparameter values are given in Appendix A, Table A1. Classification performance was evaluated using a participant-wise, leave-two-out stratified cross-validation procedure, in which two participants (stratified to preserve group balance) were held out as the test set on each fold while the model was trained on the remaining participants; this was repeated across all cross-group pairs until each pair had served in the test set exactly once—equivalently, until each participant had served in the test set 25 times—yielding 625 folds per pairwise comparison (25 × 25, corresponding to all possible pairings of one held-out participant from each of the two 25-participant groups). Each participant therefore contributed 16 × 25 = 400 trial-level test predictions. Classification was performed using both the full set of 12 differential (crime-relevant minus control) eye-tracking metrics and an optimized feature-selected subset containing only the best-performing features for each classification. Feature selection for each of the three group-pair classifications was assessed using recursive feature elimination with cross-validation (RFECV) with a logistic regression base estimator and L2 regularization, applied within the same participant-wise, leave-two-out stratified cross-validation. Within each fold, RFECV selected the subset of the 12 differential eye-tracking features that optimized cross-validated classification performance for that fold. Because several metrics are conceptually related (e.g., the dwell- and duration-based measures), feature redundancy was addressed within the classification pipeline rather than by removing correlated predictors a priori. The L2-regularized logistic-regression estimator accommodates correlated predictors by distributing coefficient weight among them, thereby reducing sensitivity to multicollinearity. RFECV then provided a data-driven mechanism for eliminating redundant or non-informative features on a fold-by-fold basis, retaining only those that contributed most to classification performance. For each feature, a selection frequency was computed as the percentage of the 625 folds in which that feature was retained in the selected subset. Higher selection frequency indicates a feature that was more consistently useful for discriminating between the two groups across resampled training sets. All data preprocessing steps including missing-value imputation, z-score normalization, RFECV feature selection were carried out strictly within the training partition of each leave-two-out fold: imputation values and normalization parameters were estimated using the training participants only and then applied unchanged to the held-out participants; feature selection and model optimization likewise had no access to the held-out participants. Consequently, no information from the left-out test participants entered any preprocessing or model-fitting stage. Ninety-five percent confidence intervals for all performance metrics were obtained by bootstrap resampling. The significance of each pairwise classification was additionally assessed with a participant-level label-permutation test: in each of 1000 iterations, class labels were randomly reassigned at the participant level (all trials of a given participant received the same permuted label, preserving the within-participant dependency structure); the participant-level logistic-regression classification on the 12 differential features was re-run; and the permuted accuracy was compared with the accuracy obtained under the true labels. The permutation *p*-value was computed as *p* = [Σ I(acc_perm ≥ acc_true) + 1]/(N + 1), with N = 1000. Performance metrics were assessed at the trial level as well as the participant level. The Trial-Level Accuracy reflects the baseline classification accuracy achieved at the level of the individual trial, prior to any aggregation of decisions to the participant level. The Participant-Level Accuracy was derived using a majority-decision rule: a participant was considered correctly classified if the model classified more than 50% of that participant’s trials correctly.

**Figure 2:**
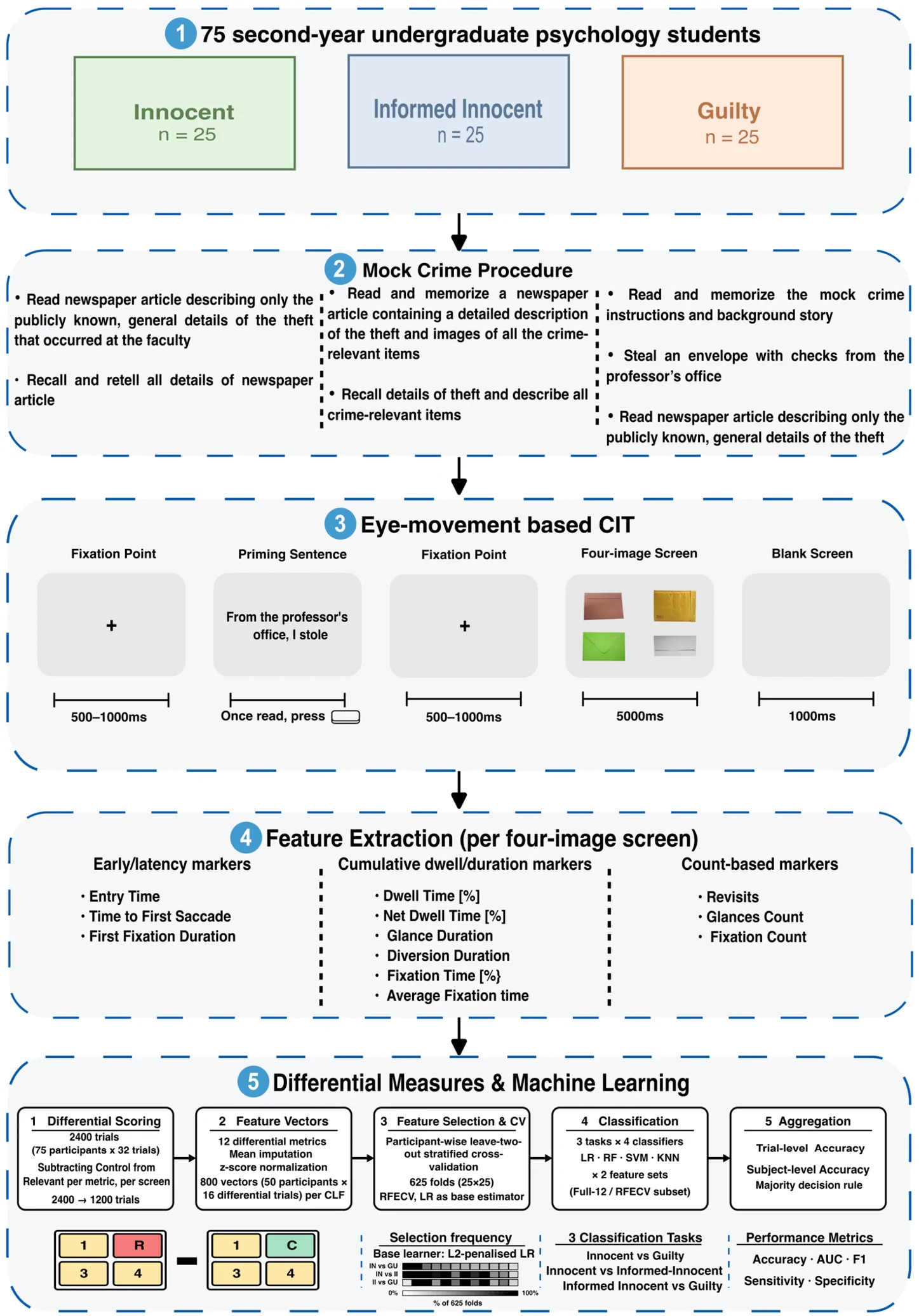
A graphical illustration of the methodological framework of this study. CLF—classifier; CV—cross-validation.

Statistical analyses were conducted using JASP 0.97, and the implementation of the classification was performed via Spyder 6.1.5 in Python 3.12 programming language, using the scikit-learn library. The methodological framework of this study is illustrated in Figure 2.

## 3. Results

### 3.1. Eye-Tracking Metrics

Table 2 presents the descriptive statistics for each of the 12 eye-tracking measures across the three experimental groups (*Innocent*, *Informed Innocent*, *Guilty*) and both item categories (crime-relevant and control).

Across conditions, participants in the *Informed Innocent* group exhibited the longest mean glance and diversion durations, as well as the highest fixation counts and dwell/fixation time percentages (e.g., Net Dwell Time: M = 34.66%). In contrast, the *Innocent* (means ranging from 22.0% to 23.3%) and *Guilty* groups (means ranging from 22.6% to 24.0%) performed similarly to one another across these metrics. Entry Time and Time to First Saccade were shortest in the *Informed Innocent* group. Compared to control stimuli, crime-relevant items elicited longer glance, diversion, and first fixation durations, as well as higher dwell time percentages. Furthermore, crime-relevant items were characterized by shorter times to the first saccade than control stimuli. The *Informed Innocent* group exhibited the greatest standard deviations across most measures, indicating substantially greater inter-individual variability within this condition. Furthermore, the SDs for crime-relevant items were markedly larger than those for control items across the majority of measures, revealing much greater variance in participant engagement with crime-relevant stimuli. These patterns indicate that visual attention was differentially allocated to crime-relevant versus control stimuli and that the eye-movement profile of the *Informed Innocent* group diverged from those of both the *Innocent* and *Guilty* groups.

### 3.2. Differences Between Innocent, Informed Innocent and Guilty Group in Eye-Tracking Metrics

A one-way MANOVA was conducted to test whether the 12 differential scores, considered jointly, differed as a function of Group (*Innocent*, *Informed Innocent*, *Guilty*), followed by 12 univariate one-way ANOVAs and Šidák-corrected pairwise post hoc comparisons for each measure individually. The multivariate effect of Group on the set of 12 differential eye-tracking measures was statistically significant, Pillai’s V = 0.821, F(24, 124) = 3.60, *p* < 0.001, indicating that the overall pattern of relevant-minus-control differences varied significantly across the *Innocent*, *Informed Innocent*, and *Guilty* groups. As shown in Table 3, univariate ANOVAs showed a significant group effect for 10 of the 12 differential measures, whereas no significant group differences were detected for Glances Count and Revisits. Effect sizes ranged from small-to-moderate (ω^2^ = 0.18–0.21 for Entry Time, First Fixation Duration, and Time to First Saccade) to very large (ω^2^ = 0.58–0.59 for Glance Duration, Diversion Duration, Net Dwell Time, Dwell Time, and Fixation Time).

Across all 10 significant measures, the post hoc comparisons revealed a highly consistent pattern, indicated in Table 3 by the superscript letters (shared letter = no significant difference): the *Innocent* and *Guilty* groups did not differ significantly from one another, and both diverged significantly from the *Informed Innocent* group. Compared to the *Innocent* and *Guilty* groups, the *Informed Innocent* group demonstrated significantly higher, positive differentials on all duration- and dwell-based metrics. For Entry Time and Time to First Saccade, the *Informed Innocent* group instead showed a substantial negative differential, reflecting faster orientation and first fixation toward crime-relevant than control items.

### 3.3. Feature Selection

**Figure 3:**
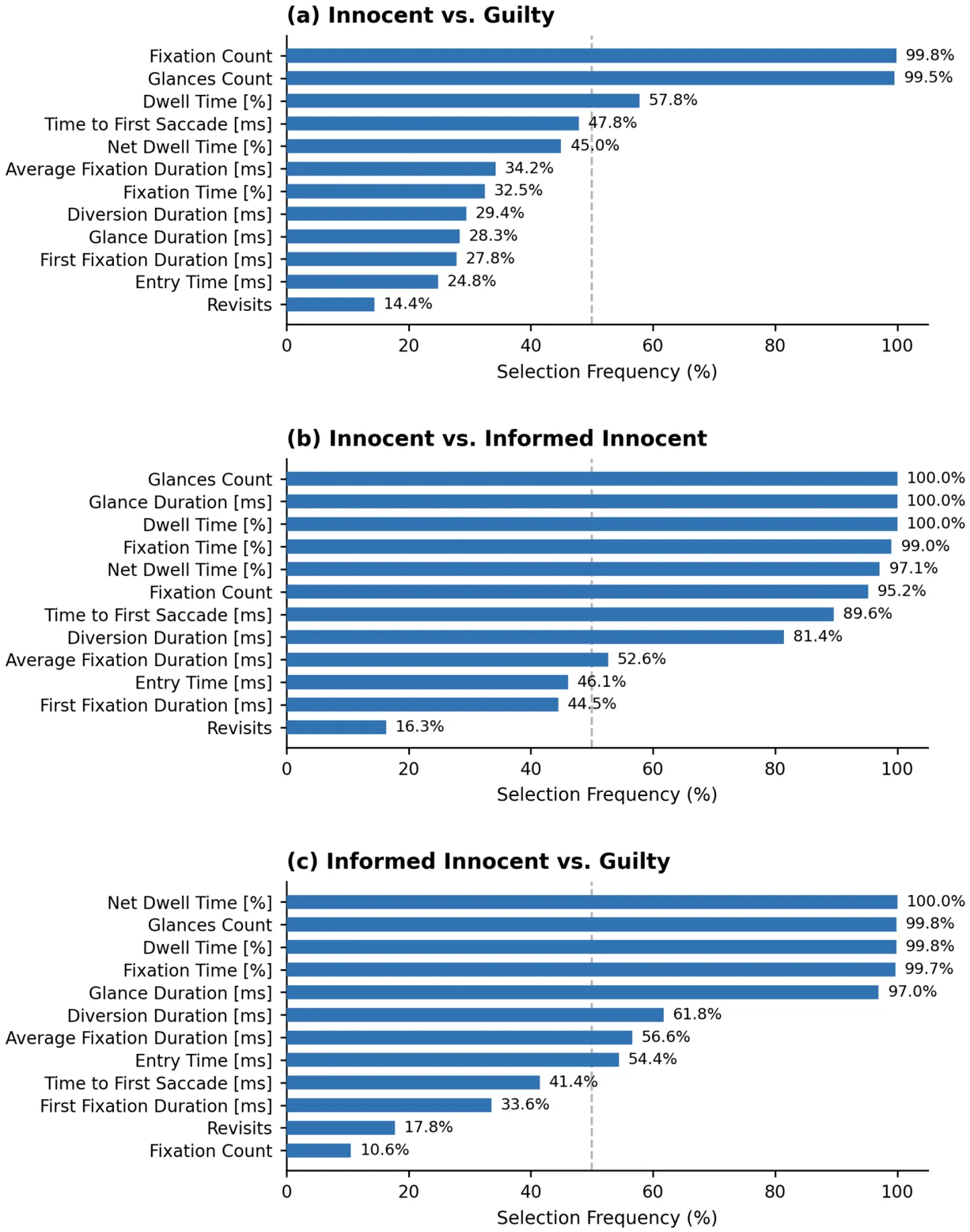
RFECV feature selection frequency (%) across 625 leave-two-out folds by group–pair classification.

Selection frequencies for all twelve differential eye-tracking features, separately for each of the three classifications, are shown in Figure 3.

Two classifications involving the *Informed Innocent* group (vs. *Innocent*, vs. *Guilty*) converge on almost the identical largely overlapping set of features. Net Dwell Time, Dwell Time, Fixation Time, Glance Duration, and Glances Count were selected in nearly every fold for both classifications (≥97%). However, Fixation Count was selected in only 10.6% of folds for *Informed Innocent* vs. *Guilty*, but it was selected in the substantial majority of folds for *Innocent* vs. *Informed Innocent* (95.2%). For the four dwell- and duration-based measures (Net Dwell Time, Dwell Time, Fixation Time, and Glance Duration), this high selection frequency is consistent with ANOVA results, where these measures had the largest effect sizes (ω^2^ = 0.58–0.59) for the *Informed Innocent* group’s distinctive differential signature. Glances Count, by contrast, was frequently retained by the feature selector despite a negligible univariate effect (F = 0.29, *p* = 0.752). Diversion Duration and Time to First Saccade showed moderate-to-high selection frequency for *Innocent* vs. *Informed Innocent* (81.4% and 89.6%, respectively) but lower frequency for *Informed Innocent* vs. *Guilty* (61.8% and 41.4%).

The feature profile for *Innocent* vs. *Guilty* was significantly different. The two most consistently selected features were Fixation Count (99.8%) and Glances Count (99.5%), while every dwell- and duration-based measure that dominated the other two classifications fell to substantially lower selection frequency. No feature other than Fixation Count and Glances Count was selected in more than 58% of folds for this classification.

### 3.4. Classification Analyses

Two levels of classification accuracy, Trial-Level Accuracy and Participant-Level Accuracy, are reported in Table 4. For each classification task, the second-named group was coded as the positive class. Sensitivity therefore reflects the true positive rate for the positive (second-named) group, and Specificity reflects the true negative rate for the first-named (reference) group, at both the trial and participant levels.

In distinguishing *Innocent* from *Guilty* participants, classification performance using both the full feature set and the optimized subset remained near chance levels across all four classification algorithms at both the Trial and the Participant levels, with LR showing the strongest but still modest separation. For the highest-performing LR model using the full feature set, Participant-level specificity reached 76% (correct identification of *Innocent* participants), whereas sensitivity was restricted to 56% (correct identification of *Guilty* participants). This discrepancy indicates that even the most robust model was considerably more effective at clearing *Innocent* participants than at detecting actual *Guilty* individuals. The other three algorithms showed a pronounced asymmetry between sensitivity and specificity: KNN with the optimized feature subset achieved participant-level specificity of 100% (correctly clearing every *Innocent* participant) but sensitivity of only 4% (correctly identifying almost no *Guilty* participants), and RF and SVM showed a similar but less extreme pattern. This indicates that when using the feature-selected subset, most algorithms defaulted toward classifying participants as *Innocent* rather than genuinely discriminating between the two groups, reinforcing that *Guilty* participants’ differential eye-movement profiles remained largely indistinguishable from *Innocent* participants’ profiles even after feature selection.

In contrast, for *Innocent* vs. *Informed Innocent*, classification using both the full feature set and the optimized subset performed well above chance for all four classification algorithms (accuracy = 72.4–76.9%, AUC = 78–85.2%) both at the trial level and, more substantially, at the participant level. LR and RF achieved the highest participant-level accuracy (92% each), and LR achieved the highest trial-level accuracy (76.9%) and AUC (85.2%). At the participant level, correct identification of *Innocent* participants (96–100%) was consistently higher than correct identification of *Informed innocent* participants (76–88%) across algorithms using all features and the optimized subset. This indicates that the classifiers were more reliable at correctly clearing naive *Innocent* participants than at correctly detecting *Informed innocent* participants, though overall separability between the two groups remained high.

For *Informed Innocent* vs. *Guilty*, classification also performed consistently above chance whether the full-feature set or the optimized feature-selected subset was utilized, with the trial-level accuracy ranging from 70.6% to 73.4% (AUC = 75.4–80.8%) and the participant-level accuracy ranging from 80% to 88% (AUC = 80–88%). RF achieved the highest participant-level accuracy (88%), while LR achieved the highest trial-level accuracy (73.4%) and AUC (80.8%). At the participant-level based on full set of features, correct identification of *Guilty* participants was identical across all four algorithms (92%), whereas correct identification of *Informed Innocent* participants was more variable and consistently lower (68–84%), with RF showing the highest specificity (84%) and correspondingly the highest overall accuracy. In classifications using the optimized feature-selected subset, three of the four algorithms (KNN, RF, and LR) converged on identical performance (accuracy = 84%, AUC = 84%, sensitivity = 92%, specificity = 76%), while SVM showed slightly lower accuracy (82%) driven by lower specificity (72%). This pattern suggests that while all four classifiers reliably identified *Guilty* participants, their performance differed mainly in how often they mistakenly classified *Informed Innocent* participants as guilty.

While feature selection maintained relatively stable trial-level accuracy and AUC metrics across all three classifications (varying by only 1–2%), it introduced substantial variability to participant-level performance in the *Innocent* vs. *Guilty* classification. Specifically, both the KNN and RF models suffered a clear decline in participant-level accuracy—dropping from 58% and 52% to 52% and 48%, respectively—due to a reduction in classification sensitivity. In contrast, the performance metrics for the other two classifications remained highly consistent regardless of whether the full-feature set or the optimized feature-selected subset was utilized. Across all three group–pair classifications, no single algorithm was uniformly superior. LR provided the strongest or near-strongest trial-level performance, while LR and RF were the strongest participant-level performers depending on the classification; KNN and SVM generally performed somewhat less well, though differences among algorithms were modest relative to the differences among classifications. Taken together, these classification results align with the trends observed in the univariate and multivariate analyses of the differential eye-tracking measures. While machine learning classifiers trained on gaze-based features easily distinguished the *Informed Innocent* group from the other two groups, the *Innocent* and *Guilty* groups remain difficult to distinguish from one another using the same feature set. To quantify the reliability of these estimates, bootstrap 95% confidence intervals for all performance metrics calculated via 1000 bootstrap iterations on the test predictions are reported in Table 5 at both the trial and participant levels across all algorithms and classification tasks. Consistent with the point estimates, the intervals for the two comparisons involving the *Informed Innocent* group lay well above chance, whereas those for *Innocent* vs. *Guilty* included or bordered chance-level performance. We performed participant-level label permutation tests (1000 iterations) using logistic regression on all 12 differential features. Under the true labels, the model reached 92% accuracy for *Innocent* vs. *Informed Innocent*, 86% for *Informed Innocent* vs. *Guilty*, and 66% for *Innocent* vs. *Guilty*. No permutation matched or exceeded the true accuracy in the first two comparisons (both *p* = 0.001), whereas 22 of the 1000 permutations did so for *Innocent* vs. *Guilty* (*p* = 0.023). This latter effect provides limited evidence for above-chance classification, as its 95% confidence interval included the chance level, and it did not survive Bonferroni correction across the three comparisons (α = 0.017), consistent with our conclusion that *Innocent* and *Guilty* participants are, for practical classification purposes, difficult to distinguish.

## 4. Discussion

### 4.1. Recognition Without Concealment: The Eye-Movement-Based CIT’s Diagnostic Validity

The present study examined which eye-tracking measures effectively differentiate individuals who recognize crime-relevant information from those who do not, while testing whether the motivation to conceal that recognition to avoid detection compromises the diagnostic validity of the test. Cumulative dwell- and duration-based metrics (Net Dwell Time, Dwell Time, Fixation Time, and Glance Duration, each selected in ≥97% of cross-validation folds) produced the largest group effects and dominated the discriminative signal, alongside glance and fixation counts. In contrast, early markers like time to first saccade and first fixation duration contributed to a lesser extent. That recognition manifested in gaze behavior is consistent with the broad literature showing that memory automatically shapes viewing behavior [12,13] and with previous eye-movement-based CIT studies reporting reliable differences between recognized and unrecognized items [14,15,16,17,18,19]. In our data, this is seen most clearly in the *Informed Innocent* group, which held crime-relevant knowledge but had no reason to hide it and which produced the most distinctive eye-movement signature of the three groups. The *Informed Innocent* group allocated markedly more and longer Dwell Time, produced more and longer fixations, and oriented faster to crime-relevant than to control items. This pattern was captured with large effect sizes (ω^2^ = 0.58–0.59 for the dwell- and duration-based measures) and was classified against genuinely naive *Innocents* with participant-level accuracies of up to 92%. This is the classic preferential-viewing, recognition-memory pattern: familiar or personally significant stimuli are prioritized by the oculomotor system, attracting earlier and more sustained fixation [13,19,20]. The negative Entry Time and Time to First Saccade differentials indicate an early reflexive orientation toward the item that completed the primed sentence, consistent with the orienting-response account of the CIT, in which personally significant items automatically capture attention [7,8]. The large inter-individual variability we observed within the *Informed Innocent* group, whose standard deviations were the largest across nearly all measures, suggests that when participants are not instructed to regulate their gaze, the physiological expression of recognition in eye movements varies widely between individuals. Taken together, these results demonstrate that in the absence of concealment motives, this novel eye-movement-based CIT, as intended, operates effectively as a direct measure of recognition memory.

### 4.2. Motivated Concealment Can Suppress Eye-Movement Recognition Responses

While the eye-movement-based CIT successfully differentiated knowledgeable but non-concealing participants from naive participants and also distinguished knowledgeable concealing participants from non-concealing ones, it failed entirely to discriminate between the *Guilty* and *Innocent* groups. Classifiers achieved strong participant-level performance whenever the *Informed Innocent* group was one of the two classes being distinguished (accuracy = 80–92% for *Innocent* vs. *Informed Innocent* and *Informed Innocent* vs. *Guilty*, across all four algorithms) but performed at only marginally above-chance levels for *Innocent* vs. *Guilty* (participant-level accuracy = 48–66%). This suggests that eye-movement recognition responses of crime-related details, reflected in cumulative dwell- and duration-based metrics, can be successfully suppressed by top-down voluntary gaze modulation as a consequence of avoiding detection in a free-viewing, object-based eye-movement CIT to the point of becoming indistinguishable from an *Innocent* examinee. Despite possessing first-hand episodic knowledge of the crime-relevant items, the *Guilty* group produced differential scores (computed relative to control items) clustered around zero and were statistically indistinguishable from those of the truly naive *Innocent* group. Consequently, classifiers trained to separate *Innocent* from *Guilty* individuals performed at or near chance (participant-level accuracy 48–66%; trial-level AUCs of 46–52.6%), whereas the same feature set separated the *Informed Innocent* group from both other groups well above chance. Revisiting our prediction that groups could be differentiated based on the presence or absence of crime-relevant knowledge, the obtained pattern of results did not fully align with our initial expectations. While knowledgeable participants were expected to display clear recognition via differential responses to critical versus control stimuli compared to naive innocent participants, this prediction was only partially supported by the data. While recognition was reliably manifested by knowledgeable non-concealing participants (the *Informed Innocent* group), it was completely attenuated in the *Guilty* group when knowledgeable participants were motivated to avoid detection. This was contrary to our research expectation that knowledgeable participants motivated to conceal their knowledge would only partially reduce their detectability relative to equally knowledgeable but non-concealing participants. Results show that under the motivation to conceal, guilty participants yielded differential scores that clustered around zero and were statistically indistinguishable from those of naive innocent participants. Therefore, the motivation to avoid detection fully moderated the expression of recognition.

For *Innocent* vs. *Guilty*, by contrast, the only features that retained any residual discriminative value between *Innocent* and *Guilty* individuals were the two count-based measures, Fixation Count and Glances Count, which were selected in 99.5–99.8% of folds. However, Glances Count failed to reach statistical significance in the univariate analyses. The dwell- and duration-based measures that dominated the other two classifications collapsed to low selection frequency here. Duration-based measures, indexing how long one looks, are under direct and continuous voluntary control, because one can simply choose to look away; by contrast, the number of discrete fixations and glances reflects a more automatic behavior of the oculomotor system that may be harder to consciously regulate.

The central novelty and primary contribution of this study stem not from advancing raw classification performance beyond existing eye-tracking paradigms but rather from demonstrating a specific operational vulnerability inherent to free-viewing, object-based CIT designs. Specifically, our findings demonstrate that recognition-related gaze responses can be deliberately suppressed through top-down voluntary gaze control to evade detection. Two distinct mechanisms that distinguish an involuntary orienting response from voluntary inhibition-related processes [7,8] offer a potential explanation. In the *Informed Innocent* group, no inhibitory demand was placed on the examinee, so the orienting response was expressed unopposed and generated the strong signature. In the *Guilty* group, the same orienting tendency was presumably elicited, but examinees were explicitly motivated to conceal recognition, and they were free to deploy their gaze as they wanted and leverage top-down oculomotor control to counteract it during a five-second unconstrained viewing window. It is well established that the human oculomotor system supports this type of voluntary control over automatic orienting behaviors. Humans can suppress reflexive, stimulus-driven saccades and voluntarily redirect gaze on instruction [24]. Our results indicate that this capacity extends to spontaneous, self-generated concealment of recognition toward realistic object stimuli.

Two design characteristics of our experimental paradigm likely maximized the efficacy of this voluntary control. First, the free-viewing instruction imposed no secondary task; examinees were simply asked to look at the display, leaving the allocation of gaze almost entirely to their autonomy without external constraints, rules, or instructions. Second, the relatively long five-second presentation window provided sufficient time for participants to redistribute fixations away from the crime-relevant item and toward neutral items after any initial capture. Under these conditions, the cumulative, whole-trial measures that carried most of the signal, such as Dwell Time, Glance Duration, and Fixation Time, are precisely the measures most susceptible to voluntary regulation, because they integrate over a window long enough for compensatory looking to erase an early bias. It is therefore plausible that an involuntary orienting toward the probe still occurred in the first few hundred milliseconds of each trial in the *Guilty* group, as has been reported for familiar faces, where an initial orienting precedes deliberate avoidance [19,31], but that it was masked by our aggregation over the full trial.

### 4.3. Comparison with Previous Studies

The findings of the present study support the general lesson from autonomic and P300 CIT research that any measure under partial voluntary control can be modulated by a motivated individual and that countermeasure resistance must be demonstrated rather than assumed [9,10,11]. Gaze direction is under fine-grained voluntary control, so it is unsurprising that a free-viewing design proved highly vulnerable.

Our results contrast instructively with the face-recognition CIT literature, in which adding a short-term memory or search task renders the test comparatively robust to voluntary manipulation, because an involuntary early orienting toward the familiar face and a subsequent overt avoidance of it still leak through despite concealment instructions [19,20,31]. The critical difference in our paradigm is the absence of any such task: with no memory or search demand, examinees had the degrees of freedom to equalize their viewing, and the signature vanished.

Our results align with recent machine learning work by Foucher and colleagues [32], who found that concealing versus revealing could be classified at roughly 74% but that adding a faking condition drove three-way accuracy down toward chance, precisely because concealing and faking induce similar, strategically managed eye-movement patterns. In both their data and ours, once examinees actively manage their gaze, the discriminability of deceptive intent collapses.

The findings in the present study diverge from a recent study by Celniak and colleagues [14]. Using a parallel-presentation, mock-crime eye-movement-based CIT paradigm methodologically similar to our own, those authors reported high discriminative accuracy (roughly 95% via their optimal classifier) between naive innocent and motivated guilty participants. In contrast, the equivalent comparison between naive and concealing conditions in our dataset yielded near-chance performance. This divergence in outcomes may be reasonably attributed to several key differences in experimental design.

First, the most diagnostic features in the earlier study were cumulative dwell measures aggregated over a longer (7.5 s) viewing window together with baseline-corrected pupil diameter. Due to the inability to standardize confounding visual factors (ambient lighting, display illumination, and stimulus brightness) pupillometry data were not analyzed in the current study. Since participants completed the experimental task at varying times of the day, it was not technically feasible to fully standardize the ambient lighting conditions in the testing room. Pupil metrics are difficult to modulate intentionally, as personally significant probes elicit pupillary responses even when an individual attempts voluntarily to divert their gaze. Thereby, the inclusion of these ocular metrics more resistant to top-down modulation likely contributed to the higher classification accuracy reported in the previous study.

Second, the previous study implemented a repetitive design wherein all trials were presented three times and subsequently aggregated, whereas in the current study we presented trials only once, without trial aggregation. Averaging across trials typically improves the signal-to-noise ratio by reducing trial-by-trial variability. In the present study, we reduced noise by calculating differential metrics, specifically subtracting control stimulus trials from relevant stimulus trials, whereas Celniak and colleagues calculated differential measure of target AOI value and median value of Non-target AOIs.

Third, while our unconstrained free-viewing paradigm instructed participants to simply look at the screen, their protocol mandated that participants inspect and familiarize themselves with all four stimuli. This design likely enhanced orienting responses toward the probe and restricted the natural gaze equalization facilitated by our unconstrained free-viewing paradigm. This interpretation aligns with their findings, where the guilty group exhibited increased, rather than decreased, fixation durations on relevant items.

Fourth, the composition of the participant samples may account for the observed differences in voluntary control. Participants in the current study were second-year undergraduate psychology students who were highly habituated to experimental settings. Their educational background coupled with familiarity with the participant role likely enhanced their ability to execute deliberate gaze manipulation. In contrast, the sample recruited by Celniak et al. was more heterogeneous, aged 19–60 years, and included non-student employees whose lack of specialized experimental familiarity may have limited deliberate modulation.

### 4.4. Methodological and Forensic Implications

The *Informed Innocent* group is not merely a methodological control; it represents a highly realistic and critical challenge in forensic practice. Crime-relevant details often leak to innocent individuals through media coverage, interrogation, or rumor, and such individuals will, as our data show, produce a strong recognition signature that an eye-movement-based CIT may misinterpret as evidence of guilt [25]. While eye-movement-based CITs hold considerable promise [23], evaluating their readiness for real-world forensic applications requires further empirical work on resistance to information leakage and voluntary gaze adjustments. Such research must incorporate larger samples, higher-stakes, ecologically valid paradigms, and direct demonstrations of countermeasure resistance. From an applied perspective, findings of this study provide several critical insights for developing a more robust eye-movement-based CIT.

First, the diagnostic architecture of the test should preferentially rely on metrics that are least susceptible to deliberate modulation, including pupillometric responses [14,17,32] under properly controlled luminance, oculomotor inhibition and microsaccade dynamics [15], and high-resolution first-saccade latency.

Second, the test should impose a concurrent task that forces processing of item identity or competes for oculomotor resources and limits the degree to which participants can freely regulate their gaze, thereby increasing diagnostic accuracy [17]. Paradigm variants that incorporated a short-term memory or recognition task, approaches that have consistently demonstrated countermeasure resistance in the face recognition literature [19,20,31] by restricting where examinees can look and driving involuntary orienting and avoidance behaviors, may offer enhanced resistance to voluntary top-down gaze modulation compared to standard detection tasks.

Third, analyses should target the temporal dynamics of gaze rather than only cumulative, whole-trial measures: the first several hundred milliseconds after stimulus onset may contain an involuntary orienting response that precedes and is not fully nullified by subsequent voluntary avoidance [19,31]. It is also important to capture early attentional responses before voluntary gaze modulation can be deployed. Shortening or segmenting the viewing window and considering rapid or sequential presentation would ensure that items are processed before gaze can be voluntarily redistributed. Measures such as the probability of a first fixation on the probe or the gaze trajectory across the first second may recover signals that aggregate dwell measures lose. Stimulus presentation parameters should be optimized to limit participants’ capacity for voluntary gaze control.

Fourth, where feasible, eye-tracking measures should be combined with autonomic or electrophysiological measures within frameworks explicitly designed to detect or resist manipulation, such as the CTP [11].

Fifth, atypical, over-regulated, or specific gaze patterns such as unnaturally uniform scanning across all stimuli should be operationalized as potential behavioral signatures of attempted manipulation [18].

### 4.5. Study Limitations and Future Directions

The present findings must be interpreted in light of several limitations. Critically, none of these limitations undermine the core conclusions of this study. Instead, they outline the boundaries of the current design and offer direct lessons for constructing more robust tests.

First, the sample was highly homogenous, consisting of 75 second-year psychology undergraduates. As psychology students, these individuals frequently participate in empirical research, are highly accustomed to laboratory conditions, and are exceptionally adept at adhering to experimental instructions. Consequently, their performance may systematically differ from that of naive lay populations in real-world forensic contexts. Our sample also fails to account for age-dependent variance in the core ocular signals upon which the test relies. Normal aging is associated with multiple sensory, attentional, oculomotor, and motor changes that may influence both the baseline eye-movement measures and the capacity for voluntary gaze control [33], so gaze-based systems should be designed and validated against the diverse demographics rather than assumed to generalize across age groups [34].

Second, the sample was modest (n = 25 per group), which limits statistical power for the classification analyses and renders participant-level estimates from leave-two-out cross-validation sensitive to the behavior of individual participants; the combination of near-perfect specificity and very low sensitivity observed for some classifiers in the *Innocent* vs. *Guilty* contrast illustrates this instability.

Third, as in all mock-crime research, ecological validity is limited. Participants faced no real stakes or consequences; the emotional arousal accompanying a genuine crime was absent; and guilt was instructed rather than self-initiated.

Fourth, guilty examinees were instructed to avoid detection, but they received no explicit instructions or coaching on how to conceal recognition, and we did not collect self-report data on how participants attempted to avoid detection by voluntarily controlling their gaze. We therefore cannot characterize whether they spread their gaze evenly, fixated neutral items, actively avoided the probe, or simply disengaged from the task, gaze manipulations that may carry different and potentially detectable signatures. Relatedly, our design cannot fully distinguish successful active countermeasure usage from mere reduced engagement. Both may produce the near-zero differentials we observed, yet they differ in theoretical meaning.

Fifth, pupillometry, probably the eye-tracking metric least susceptible to voluntary control, was excluded because ambient lighting, monitor illumination, and stimulus brightness could not be held constant.

Sixth, the SMI Experiment Center software did not permit trial randomization, so trial order, although quasi-randomized, was fixed and identical across participants, leaving potential sequence, practice, and fatigue effects uncontrolled.

Seventh, several of the twelve measures are strongly collinear (for example, Dwell Time, Net Dwell Time, and Fixation Time; and Glance and Diversion Duration), so the feature set does not constitute twelve independent dimensions.

Finally, visual and cognitive complexity were matched within but not across crime-relevant categories, introducing between-category variance into the differential scores.

Future studies should directly assess the specific gaze modulation strategies deployed by participants. For example, through post-test interviews, contrasting instructed countermeasure conditions directly against spontaneous concealment, or trial-by-trial analyses of gaze regulation, so that these gaze-control strategies can be measured directly.

Furthermore, to mitigate the efficacy of voluntary gaze control, future research should investigate task variants that structurally restrict an examinee’s opportunity to manipulate their gaze: incorporating concurrent working memory or recognition tasks to deplete cognitive resources, manipulating spatial and temporal stimulus presentation (e.g., sequential versus parallel displays, or rapid serial visual presentation) to systematically restrict the examinee’s capacity for voluntary gaze equalization or using pupillometric and microsaccadic measures.

Applying machine learning models to features pre-selected for their inherent resistance to manipulation would further clarify the extent to which the cognitive recognition signal can be recovered when examinees are highly motivated to conceal it.

Given that no single modality proved immune to countermeasures, there is a clear need to systematically evaluate multimodal protocols that integrate eye-tracking metrics with autonomic or electrocortical indices within countermeasure-resistant designs.

An incentivized study, in which examinees are explicitly trained in countermeasures and rewarded for evading detection, would provide a significantly more rigorous test of robustness than the spontaneous concealment examined here.

Future studies must replicate these findings using larger, more demographically heterogeneous samples. Specifically, research should evaluate older adults and broader general populations spanning a wide age spectrum with diverse visual, oculomotor, and cognitive capabilities.

## 5. Conclusions

Using a newly developed free-viewing, parallel-presentation eye-movement-based CIT administered to naive Innocent, Informed Innocent, and Guilty individuals, we found that crime-relevant knowledge produces a strong and classifiable eye-movement signature, comprising longer dwell and fixation times and faster orienting toward crime-relevant items, but only when the individual has no motive to conceal it. When knowledgeable individuals were motivated to avoid detection, they voluntarily modulated their gaze so effectively that their eye-movement profiles became statistically indistinguishable from those of genuinely innocent individuals, driving classification performance to chance. The primary contribution of this study thus lies in demonstrating this operational limitation: voluntary control of eye movements can substantially reduce the discriminative power of a free-viewing eye-movement-based CIT. Developing a resilient test will require designs that constrain voluntary eye-movement control. This can be achieved by implementing concurrent memory load, brief or rapid stimulus presentation, or visual search tasks that constrain gaze, and reliance on markers that are least susceptible to voluntary control such as pupillary measures. Optimally, these behavioral methods should be combined with physiological and electrocortical measures within protocols explicitly developed to detect or resist manipulation. Prioritizing resistance to voluntary gaze modulation as a core design criterion, rather than a post hoc consideration, is essential for eye tracking methodology to achieve its full potential within concealed information detection paradigms.

## Figures and Tables

**Table 1:** Eye-tracking metrics: operational definitions and typically indexed processes.

Metric	Definition	Reflection
Entry Time	Duration from start of the trial to the first hit of the AOI.	Automatic orienting and attentional capture
Time to First Saccade	Time elapsed from the onset of stimulus presentation until the participant’s first detected saccade	
First Fixation Duration	Duration of the first fixation to hit the AOI	Early processing and initial encoding depth
Net Dwell Time [%]	(Sum of sample durations for all gaze data samples that hit the AOI)/(end time − start time)	Sustained visual attention and depth of processing
Dwell Time [%]	(Sum of durations from all fixations and saccades that hit the AOI)/(end time − start time)	
Fixation Time [%]	(Sum of the fixation durations inside the AOI)/(end time − start time)	
Glance Duration	Saccade duration for entering the object + sum of all fixation durations and saccade durations before the eyes begin to leave the AOI (dwell time + duration of saccade entering AOI)	
Diversion Duration	Sum of saccade durations for entering and leaving the object + sum of all fixation durations and saccade durations before the eyes begin to leave the AOI (glance duration + duration of saccade leaving AOI)	
Average Fixation Duration	Mean duration of the fixations detected within the AOI (average length of time the eyes remain relatively stable during each fixation)	
Fixation Count	Number of fixations inside the AOI	Re-inspection and visual-search behavior
Glances Count	Number of glances to a target (saccades coming from outside) within a certain period (the counter is incremented each time a fixation hits the AOI, if not hit before).	
Revisits	Number of glances − 1	

Note. The process labels in Table 1 reflect the interpretations most commonly attributed to each measure in the literature; they are not processes that can be isolated within the present experimental paradigm.

**Table 2:** Descriptive statistics for eye-tracking measures by group and type of items.

Eye-Tracking Measure	Innocent M (SD)	Informed Innocent M (SD)	Guilty M (SD)	Relevant Items M (SD)	Control Items M (SD)
Entry Time (ms)	1041 (347.2)	687 (229.3)	987.9 (335.2)	925.9 (385)	884.8 (298.7)
Glance Duration (ms)	1158 (202.5)	1716 (847.2)	1192 (312.4)	1538 (772.1)	1172 (188.3)
Diversion Duration (ms)	1214 (207.8)	1773 (840.6)	1255 (317.5)	1594 (769.2)	1234 (191.6)
First Fixation Duration (ms)	361.9 (117.5)	372.2 (164)	377.3 (111.2)	414.8 (144.2)	326.1 (102.2)
Glances Count	1.9 (0.438)	2.1 (0.397)	1.9 (0.430)	1.9 (0.448)	2 (0.423)
Revisits	0.946 (0.427)	1.2 (0.382)	0.939 (0.414)	0.976 (0.427)	1 (0.411)
Fixation Count	2.776 (0.604)	3.6 (1)	2.8 (0.670)	3.2 (1.1)	2.9 (0.524)
Net Dwell Time (%)	23.33 (3.9)	34.7 (16.9)	24 (6.2)	31 (15.4)	23.7 (3.6)
Dwell Time (%)	22 (3.9)	33.1 (16.7)	22.6 (6.1)	29.6 (15.4)	22.2 (3.7)
Fixation Time (%)	21.7 (3.9)	32.6 (16.7)	22.2 (5.9)	29.1 (15.2)	21.9 (3.6)
Average Fixation Duration (ms)	423.6 (112.5)	475.8 (224.4)	422.7 (111.7)	483.9 (190.6)	397.5 (105.5)
Time to First Saccade (ms)	1563 (436.5)	1014 (348.1)	1432 (404.8)	1285 (498.6)	1388 (414.9)

Note. M = mean; SD = standard deviation.

**Table 3:** Differences between Innocent, Informed Innocent and Guilty in differential eye-tracking scores.

Eye-Tracking Differential Score	Innocent	Informed Innocent	Guilty	F(2, 72)	*p*	ω^2^
Entry Time (ms)	180.5 ^a^ (291.3)	−146.2 ^b^ (244.5)	89.6 ^a^ (254.1)	10.19	<0.001	0.197
Glance Duration (ms)	−127.2 ^a^ (255.4)	1248 ^b^ (753.2)	−23.0 ^a^ (441.4)	53.13	<0.001	0.582
Diversion Duration (ms)	−129.1 ^a^ (259.2)	1237 ^b^ (748.5)	−26.4 ^a^ (447.8)	52.41	<0.001	0.578
First Fixation Duration (ms)	38.02 ^a^ (107.7)	194.5 ^b^ (191.6)	33.8 ^a^ (103.5)	10.66	<0.001	0.205
Glances Count	−0.075 (0.271)	−0.047 (0.3987)	−0.122 (0.3821)	0.29	0.752	0.000
Revisits	−0.084 (0.294)	−0.045 (0.3762)	−0.118 (0.3804)	0.27	0.767	0.000
Fixation Count	−0.267 ^a^ (0.620)	1.3 ^b^ (0.8817)	−0.032 ^a^ (0.7417)	33.14	<0.001	0.462
Net Dwell Time (%)	−2.51 ^a^ (4.9)	24.9 ^b^ (15.1)	−0.682 ^a^ (8.792)	53.51	<0.001	0.583
Dwell Time (%)	−2.6 ^a^ (5.0)	25.0 ^b^ (15.04)	−0.406 ^a^ (8.571)	54.48	<0.001	0.588
Fixation Time (%)	−2.4 ^a^ (4.9)	24.7 ^b^ (14.9)	−0.409 ^a^ (8.419)	53.88	<0.001	0.585
Average Fixation Duration (ms)	4.5 ^a^ (110.4)	252.7 ^b^ (248.1)	2.1 ^a^ (95.84)	18.74	<0.001	0.321
Time to First Saccade (ms)	68.2 ^a^ (321.6)	−323 ^b^ (294.8)	−47.6 ^a^ (376.5)	9.12	<0.001	0.178

Note. n = 25 per group; within each row, group means that do not share a common superscript letter (a, b) differed significantly from one another (*p* < 0.05) in Šidák-corrected pairwise post hoc comparisons, whereas means sharing the same superscript letter did not differ significantly from one another.

**Table 4:** Trial-level and participant-level classification performance without and with feature selection.

Classification Without Feature Selection		Trial Level					Participant Level				
		Accuracy	AUC	Sensitivity	Specificity	F1-Score	Accuracy	AUC	Sensitivity	Specificity	F1-Score
Innocent vs. Guilty	KNN	50.1%	48.1%	48.5%	48.0%	48.4%	58.0%	58.0%	56.0%	60.0%	57.1%
	RF	50.3%	49.4%	53.7%	47.7%	52.2%	52.0%	52.0%	52.0%	52.0%	52.0%
	LR	52.9%	52.6%	52.5%	53.2%	52.7%	66.0%	**66.0%**	56.0%	76.0%	62.2%
	SVM	48.8%	47.5%	57.5%	38.5%	52.5%	50.0%	50.0%	44.0%	56.0%	46.8%
Innocent vs. Informed Innocent	KNN	72.4%	78.0%	68.2%	75.0%	70.6%	90.0%	90.0%	84.0%	96.0%	89.4%
	RF	74.6%	82.5%	70.5%	79.5%	73.8%	92.0%	**92.0%**	88.0%	96.0%	91.7%
	LR	76.9%	85.2%	70.7%	82.7%	75.3%	92.0%	**92.0%**	84.0%	100.0%	91.3%
	SVM	76.1%	84.4%	72.2%	79.0%	74.8%	88.0%	88.0%	76.0%	100.0%	86.4%
Informed Innocent vs. Guilty	KNN	70.8%	75.4%	75.5%	64.5%	71.6%	84.0%	84.0%	92.0%	76.0%	85.2%
	RF	70.6%	77.0%	76.2%	66.0%	72.5%	88.0%	**88.0%**	92.0%	84.0%	88.5%
	LR	73.4%	80.8%	80.5%	66.5%	75.2%	86.0%	86.0%	92.0%	80.0%	86.8%
	SVM	72.8%	79.3%	80.2%	66.7%	75.2%	80.0%	80.0%	92.0%	68.0%	82.1%
**Classification with feature selection**											
Innocent vs. Guilty	KNN	49.3%	47.6%	27.7%	69.2%	35.0%	52.0%	52.0%	4.0%	100.0%	7.7%
	RF	49.7%	49.0%	47.0%	53.5%	48.6%	48.0%	48.0%	28.0%	68.0%	35.0%
	LR	51.9%	51.1%	49.5%	52.2%	50.2%	60.0%	**60.0%**	60.0%	60.0%	60.0%
	SVM	50.0%	46.0%	60.0%	35.7%	53.51%	54.0%	54.0%	28.0%	80.0%	37.8%
Innocent vs. Informed Innocent	KNN	72.4%	79.2%	68.2%	75.5%	70.8%	92.0%	**92.0%**	84.0%	100.0%	91.3%
	RF	75.4%	82.9%	72.7%	80.7%	75.8%	92.0%	**92.0%**	88.0%	96.0%	91.7%
	LR	76.3%	84.9%	71.2%	81.7%	75.2%	92.0%	**92.0%**	84.0%	100.0%	91.3%
	SVM	76.2%	83.7%	73.5%	79.0%	75.6%	92.0%	**92.0%**	84.0%	100.0%	91.3%
Informed Innocent vs. Guilty	KNN	70.6%	77.3%	77.7%	65.7%	73.3%	84.0%	**84.0%**	92.0%	76.0%	85.2%
	RF	70.7%	78.0%	76.7%	66.2%	72.9%	84.0%	**84.0%**	92.0%	76.0%	85.2%
	LR	72.4%	80.4%	79.0%	64.0%	73.5%	84.0%	**84.0%**	92.0%	76.0%	85.2%
	SVM	73.2%	79.3%	80.7%	66.5%	75.4%	82.0%	82.0%	92.0%	72.0%	83.6%

Note. Trial level = accuracy at the individual-trial level; Participant level = accuracy after applying a majority-decision rule (a participant was classified correctly if more than 50% of their trials were classified correctly). KNN = k-Nearest Neighbors; RF = Random Forest; LR = Logistic Regression; SVM = Support Vector Machine. Bold formatting was used to denote the highest classification accuracy achieved for each respective comparison.

**Table 5:** Bootstrap 95% confidence intervals for classification performance by comparison and algorithm (trial and participant levels).

Comparison	Algorithm	AUC [%]	Accuracy [%]	Sensitivity [%]	Specificity [%]	F1 [%]
Trial level						
Innocent vs. Informed Innocent	KNN	[77.07, 83.21]	[69.38, 75.62]	[64.16, 73.28]	[72.20, 80.26]	[67.72, 75.10]
	LR	[82.26, 87.65]	[73.00, 79.00]	[65.92, 74.68]	[77.75, 85.06]	[71.06, 78.03]
	RF	[80.02, 85.81]	[73.25, 79.00]	[67.53, 76.32]	[76.37, 83.83]	[71.56, 78.35]
	SVM	[81.13, 86.45]	[73.75, 79.50]	[69.57, 78.04]	[75.74, 83.54]	[72.68, 79.26]
Informed Innocent vs. Guilty	KNN	[74.93, 81.52]	[69.62, 76.00]	[74.35, 82.71]	[62.56, 71.57]	[70.69, 77.84]
	LR	[77.56, 83.64]	[69.25, 75.38]	[75.74, 83.80]	[59.76, 69.40]	[70.70, 77.41]
	RF	[74.57, 81.10]	[68.88, 75.12]	[74.56, 82.76]	[60.70, 70.20]	[70.29, 77.16]
	SVM	[76.09, 82.46]	[71.00, 77.00]	[78.03, 85.19]	[61.56, 70.57]	[72.64, 79.04]
Innocent vs. Guilty	KNN	[46.37, 54.07]	[45.00, 52.12]	[26.11, 35.02]	[62.30, 71.50]	[32.53, 41.83]
	LR	[46.89, 54.85]	[47.38, 54.50]	[46.33, 55.83]	[46.04, 55.69]	[46.80, 54.96]
	RF	[45.07, 52.98]	[46.88, 53.75]	[41.48, 50.99]	[49.73, 59.36]	[43.70, 52.26]
	SVM	[42.02, 49.76]	[44.12, 50.88]	[56.41, 65.88]	[28.92, 38.40]	[49.71, 57.63]
Participant level						
Innocent vs. Informed Innocent	KNN	—	[75.80, 94.17]	[62.96, 95.24]	[80.00, 100.00]	[72.22, 94.55]
	LR	—	[83.93, 98.08]	[67.86, 96.15]	[100.00, 100.00]	[80.85, 98.04]
	RF	—	[80.68, 97.83]	[66.67, 96.30]	[86.96, 100.00]	[77.42, 97.78]
	SVM	—	[84.78, 98.28]	[69.57, 96.55]	[100.00, 100.00]	[82.05, 98.25]
Informed Innocent vs. Guilty	KNN	—	[73.34, 93.51]	[80.00, 100.00]	[59.26, 90.91]	[73.68, 94.34]
	LR	—	[73.03, 93.51]	[80.77, 100.00]	[57.14, 91.30]	[73.08, 94.44]
	RF	—	[73.35, 93.51]	[79.31, 100.00]	[58.33, 92.00]	[72.73, 93.88]
	SVM	—	[68.59, 90.58]	[77.78, 100.00]	[50.00, 86.21]	[70.00, 91.80]
Innocent vs. Guilty	KNN	—	[42.50, 57.41]	[0.00, 20.00]	[80.00, 100.00]	[0.00, 30.77]
	LR	—	[43.96, 72.00]	[40.00, 80.00]	[35.48, 75.86]	[40.00, 73.08]
	RF	—	[34.72, 61.35]	[10.71, 46.43]	[50.00, 86.36]	[13.79, 54.17]
	SVM	—	[40.71, 63.41]	[9.09, 42.31]	[63.64, 95.00]	[12.90, 52.63]

Note. AUC = area under the ROC curve; KNN = k-Nearest Neighbors; LR = Logistic Regression; RF = Random Forest; SVM = Support Vector Machine. Participant-level AUC is not reported because under the balanced majority-vote design, it equals balanced accuracy. For each comparison, the second-named group is the positive class.

## Data Availability

The datasets presented in this article are not readily available because the data are part of an ongoing study. Requests to access the datasets should be directed to the corresponding author.

## Supplementary Materials

The following supporting information can be downloaded at: https://www.mdpi.com/article/10.3390/jemr19050096/s1, Supplementary Images S1: The 32 images used in eye-movement-based CIT.

## Abbreviations

The following abbreviations are used in this manuscript:

ANOVA	Analysis of Variance
AOI	Area of Interest
AUC	Area Under the Curve
CIT	Concealed Information Test
CTP	Complex Trial Protocol
KNN	K-Nearest Neighbors
LR	Logistic Regression
MANOVA	Multivariate Analysis of Variance
RF	Random Forest
RFECV	Recursive Feature Elimination with Cross-Validation
SVM	Support Vector Machine

## Appendix A

**Table A1:** Hyperparameter values for all applied classifiers: k-Nearest Neighbors (KNN), Random Forest (RF), Logistic Regression (LR), and Support Vector Machine (SVM).

Classifier	Hyperparameter	Value
KNN	leaf_size	30
	metric	minkowski
	n_neighbors	5
	weights	uniform
RF	max_depth	9
	n_estimators	33
	random_state	None
	verbose	0
	warm_start	FALSE
LR	C	0.1
	l1_ratio	None
	max_iter	100
	penalty	l2
	random_state	None
	solver	saga
	tol	0.0001
	verbose	0
	warm_start	FALSE
SVM	C	1
	gamma	scale
	kernel	rbf
	max_iter	−1
	probability	TRUE
	random_state	None
	tol	0.001
	verbose	FALSE
